# B-type nuclear lamin and the nuclear pore complex Nup107-160 influences maintenance of the spindle envelope required for cytokinesis in *Drosophila* male meiosis

**DOI:** 10.1242/bio.017566

**Published:** 2016-07-11

**Authors:** Daisuke Hayashi, Karin Tanabe, Hiroka Katsube, Yoshihiro H. Inoue

**Affiliations:** Insect Biomedical Research Center, Kyoto Institute of Technology, Matsugasaki, Sakyo-ku, Kyoto 606-0962, Japan

**Keywords:** Nuclear lamin, Male meiosis, NPC, Cytokinesis, Drosophila

## Abstract

In higher eukaryotes, nuclear envelope (NE) disassembly allows chromatin to condense and spindle microtubules to access kinetochores. The nuclear lamina, which strengthens the NE, is composed of a polymer meshwork made of A- and B-type lamins. We found that the B-type lamin (Lam) is not fully disassembled and continues to localize along the spindle envelope structure during *Drosophila* male meiosis I, while the A-type lamin (LamC) is completely dispersed throughout the cytoplasm. Among the nuclear pore complex proteins, Nup107 co-localized with Lam during this meiotic division. Surprisingly, Lam depletion resulted in a higher frequency of cytokinesis failure in male meiosis. We also observed the similar meiotic phenotype in Nup107-depleted cells. Abnormal localization of Lam was found in the Nup-depleted cells at premeiotic and meiotic stages. The central spindle microtubules became abnormal and recruitment of a contractile ring component to the cleavage sites was disrupted in Lam-depleted cells and Nup107-depleted cells. Therefore, we speculate that both proteins are required for a reinforcement of the spindle envelope, which supports the formation of central spindle microtubules essential for cytokinesis in *Drosophila* male meiosis.

## Abstract (precis)

**Summary:** B-type lamin, positioned by nuclear pore component Nup107, strengthens the membranous intracellular structure supporting the microtubule bundles essential for cytokinesis in *Drosophila* male meiosis.

## INTRODUCTION

The nuclear envelope (NE) is a characteristic structure of eukaryotic cells and consists of two closely juxtaposed membranes, termed the outer and inner nuclear membranes (D'Angelo and Hetzer, 2008; Hetzer, 2010; Hetzer et al., 2005; Tran and Wente, 2006). The outer membrane is contiguous with the endoplasmic reticulum (ER) in the cytoplasm. The inner membrane contains a unique set of membrane proteins required for contact with chromatin and the nuclear matrix. Beneath the inner membrane is a meshwork of intermediate filaments called the nuclear lamina that contacts chromatin and plays a role in stabilizing the nuclear membranes from the inside (Gruenbaum et al., 2000, 2003). The lamina filaments covering chromatin consist of two types of lamin, A-type and B-type lamins. For example, *Drosophila melanogaster* possesses a B-type lamin, laminDm0 (Lam), which is expressed ubiquitously, whereas an A-type lamin designated lamin C (LamC) is expressed in a tissue-specific manner (Lenz-Bohme et al., 1997; Riemer et al., 1995; Schulze et al., 2009). An integral protein complex comprising transmembrane proteins is incorporated into the inner membrane, and proteins spanning the outer membrane anchoring sites for cytoplasmic cytoskeletons and nuclear lamins (D'Angelo and Hetzer, 2008).

The two nuclear membranes fuse with each other at sites of the nuclear pore complex (NPC). The NPC is a large multiprotein complex with an outer diameter of about 100 nm, embedded in the NE. It encloses an inner channel about 40 nm wide, which regulates the transport of diverse molecules between the nucleus and the cytoplasm (Lim and Fahrenkrog, 2006; Tran and Wente, 2006). A single NPC consists of ∼30 different protein components termed nucleoporins (Nups) (Antonin et al., 2008; Guttinger et al., 2009; Hetzer, 2010). The NPC is basically composed of three major parts: a central core, cytoplasmic filaments and a nuclear basket (Duheron et al., 2014). The central core, which is the major scaffold of the NPC, contains three ring-like substructures such as the nucleoplasmic ring surrounding the central channel. A subset of the Nups, referred to as the Nup170-160 complex, plays a key role as a component of the nucleoplasmic ring (Boehmer et al., 2003; Guttinger et al., 2009; Walther et al., 2001; Walther et al., 2003).

During cell divisions in higher eukaryotes (metazoans), both the NE and the NPC undergo complete breakdown at the prophase to prometaphase stage. The NE breakdown (NEBD) is widely considered as one of the most remarkable characteristics of mitosis in eukaryotic cells (Dultz et al., 2008; Terasaki et al., 2001). It allows microtubules emanating from centrosomes at spindle poles to capture chromosomes at kinetochores. Vertebrate cultured cells display so-called open mitosis, in which the whole of the nuclear membrane has completely vanished before metaphase (Dultz et al., 2008). However, there are some variant types of NEBD among eukaryotic species, and even between cell types within the same species (Sazer et al., 2014). In contrast to open mitosis, nuclear membrane integrity is always maintained throughout mitosis in yeast (Arnone et al., 2013). In addition, an intermediate type called semi-closed mitosis can be observed in syncytial nuclear division cycles of early embryos in *Drosophila* or in starfish oocytes (Harel et al., 1989; Katsani et al., 2008; Kiseleva et al., 2001; Lenart and Ellenberg, 2003). Unlike the NEs in vertebrate cells, nuclei at the syncytial stage undergo a regional NEBD only around spindle poles (Katsani et al., 2008; Kiseleva et al., 2001). A similar type of semi-closed cell division was also observed in *Drosophila* neuroblasts at the larval stage (Katsani et al., 2008).

In addition to the conventional role of nuclear envelope components as a barrier to separate genomic DNA from the cytoplasm, it has been reported that a fraction of B-type lamin is localized on spindle microtubules in mammalian cells and in cell extracts prepared from mature *Xenopus* oocytes (Georgatos et al., 1997; Ma et al., 2009). Moreover, a reduction of the B-type lamin also influenced nuclear organization, formation of spindle microtubules and mitotic progression. Depletion of this lamin affected formation of spindle microtubules in mammalian cultured cells (Liu et al., 2000; Ma et al., 2009; Tsai et al., 2006). The Nup107-160 complex, which plays a key role as the NPC scaffold, also shows kinetochore localization during mitosis and is indispensable for correct bipolar spindle assembly (Belgareh et al., 2001; Loiodice et al., 2004; Orjalo et al., 2006; Zuccolo et al., 2007). It was also identified as a component of the spindle matrix in *Drosophila* early embryos at syncytial stages, in contrast to its kinetochore localization in mammalian cells (Katsani et al., 2008). Thus, it is necessary to further examine these NE proteins using not only syncytial blastoderm embryos but also cell types that are suitable to observe.

For detailed observation of the nuclear envelope structure as well as dynamics during cell divisions, *Drosophila* primary spermatocytes offer several advantages. A spermatocyte undergoes a remarkable cell enlargement of up to 16 times in volume before meiotic entry. Thus, mature spermatocytes that have completed their cell growth stages are the largest diploid cells among all proliferative cells in *Drosophila* (Fuller, 1993; Inoue et al., 2004; Inoue et al., 2012). These cells allow us to examine NE architecture in detail. The NE undergoes a dynamic alteration in its structure according to the progression of cell divisions. The primary spermatocyte is particularly rich in membranous architecture and possesses well-developed ER networks in the cytoplasm (Inoue et al., 2012). A previous morphological study of spermatocytes using electron microscopy revealed that primary spermatocytes entering meiosis I maintained a rigid membrane system developed at the premeiotic stage (Fuller, 1993). In the first meiotic division, the multilayered membranes surrounding the primary spermatocyte nucleus are maintained until at least anaphase I and disappeared prior to telophase I (Church and Lin, 1982; Fuller, 1993). By contrast, it was unclear whether the secondary spermatocyte nucleus displayed similar NE dynamics. Instead of electron microscope observation, the multilayered NE was recently visualized using ER-enriched proteins as markers (Kitazawa et al., 2012). The round-shaped NE at metaphase transformed into an oval-shaped architecture termed the spindle envelope (Bobinnec et al., 2003; Dorogova et al., 2009; Inoue et al., 2004). Within the space surrounded by the multilayered NE, segregation of sets of paired homologous chromosomes is carried out. This NE structure is considered to play an important role in supporting the formation of spindle microtubules (Kitazawa et al., 2012).

In this study, we investigated the dynamics of lamins during the first and second meiotic divisions in *Drosophila* males. We found that Lam disassembled only around the spindle poles before metaphase I and continued to be localized along the spindle envelope structure during meiosis I, while LamC was completely dispersed throughout the cytoplasm. Nup107 displayed co-localization with Lam during the first meiotic division. Spermatocyte-specific depletion of Lam and of Nups comprising the Nup107-160 complex resulted in a failure of cytokinesis at a higher frequency. Our observations suggest that Lam and Nup107 play a role in reinforcing the nuclear envelope and spindle envelope structures during male meiosis I. The envelope structures facilitate the formation of central spindle microtubules that are essential for cytokinesis. Therefore, we propose that Lam and the Nup107-160 complex are required for the formation and reinforcement of the nuclear envelope and its derivatives in *Drosophila* male meiosis.

## RESULTS

### Two types of nuclear lamin are closely associated with each other under the NE in premeiotic spermatocytes

Primary spermatocytes from *D. melanogaster* express both the B-type Lam and the A-type LamC. *In situ* PLA signals between the two proteins were detected in nuclei of premeiotic spermatocytes (Fig. S1A). Thus, these two lamins are closely associated with each other (within 40 nm). The signals were also observed in sister nuclei newly constructed at telophase I (Fig. S1B). We showed that expression of their dsRNAs could effectively but partially deplete each protein (Fig. S2). Lam depletion caused a loss of Lam and LamC from the nuclear envelope (Fig. S3A-A″,C). Conversely, LamC depletion did not influence the nuclear localization of Lam (Fig. S3B-B″). These data suggest that LamC localization depends on the presence of Lam in the nuclear lamina of premeiotic nuclei.

**Fig. 1.**
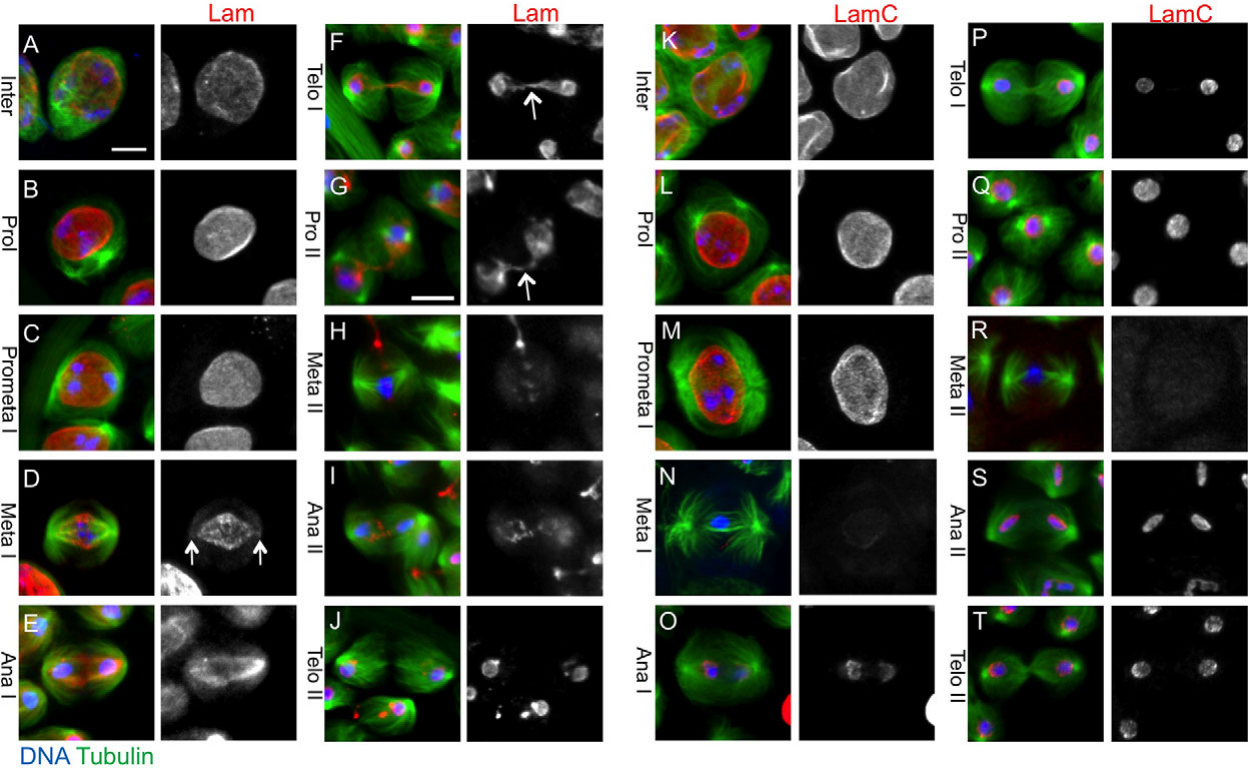
**Immunolocalization of Lam and LamC during meiosis I and II in *Drosophila* males.** (A) Immunostaining of premeiotic spermatocytes with anti-Lam antibody. (B-F) Anti-Lam immunostaining of spermatocytes undergoing meiosis I. (G-J) Anti-Lam immunostaining of meiosis II cells. (K) Anti-LamC immunostaining of premeiotic spermatocytes. (L-P) Anti-LamC immunostaining of meiosis I cells. (Q-T) Anti-LamC immunostaining of meiosis II cells. Green: GFP-β-Tubulin; blue: DNA; red: anti-Lam staining in A-J, anti-LamC staining in K-T. Lam is distributed along the spindle-shaped NE. The NE seems to be broken down only around the spindle poles (D, arrows) so that spindle microtubules can elongate into the nuclear space surrounded by the NE. Note that Lam remains localized during later stages of meiosis I (F,G, arrows), while the LamC signal disappears at metaphase I. Scale bar: 10 μm.

**Fig. 2.**
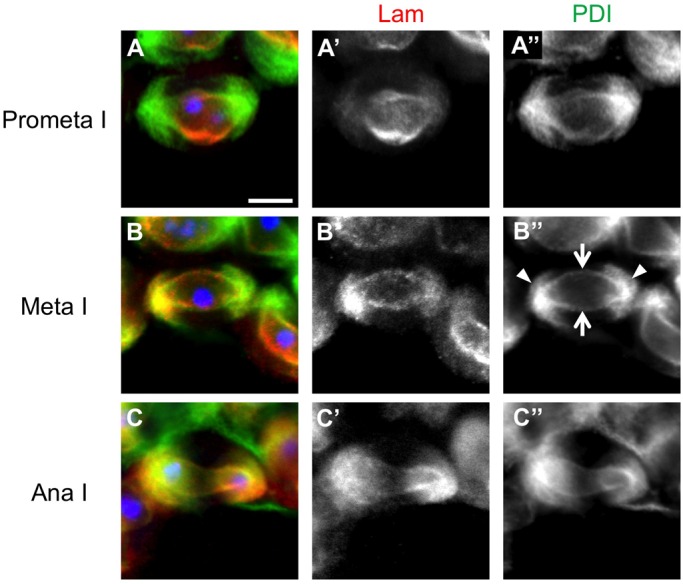
**Anti-Lam immunostaining of meiosis I cells expressing PDI-GFP as a marker for the NE and its derivative, the spindle envelope.** A primary spermatocyte from a meiotic cyst at prometaphase I (A), metaphase I (B) or anaphase I (C). Both Lam and the ER marker (PDI) were co-localized along the spindle envelope. Red in A,B,C and white in A′,B′,C′: anti-Lam staining; green in A,B,C and white in A″,B″,C″: PDI-GFP. Arrows indicate the spindle envelope, which is rich in the ER protein. Arrowheads show the astral membrane, in which the ER protein is abundant. Note that Lam is distributed on the nuclear envelope-derived spindle envelope and astral membrane. Blue in A,B,C: DNA. Scale bar: 10 μm.

We next investigated the cellular localization of both lamins during meiotic divisions by immunostaining experiments (Fig. 1A-T). Interestingly, the two lamins showed different dynamics during the first meiotic division. These two lamins seemed to be co-localized on the NE until prometaphase I (Fig. 1A-C). Thereafter, two types of nuclear lamina, one consisting of Lam and the other of LamC, displayed different dynamics during meiotic divisions. At metaphase I, Lam was distributed on the nuclear structure in a spindle shape (Fig. 1D). To identify the intracellular structure on which the Lam was localized during meiosis I, we investigated its co-localization with several markers other than microtubules. Two characteristic cell organelles are formed in male meiosis I; the astral membrane structure is closely associated with astral microtubules, and the spindle envelope structure surrounding central spindle microtubules is derived from the NE. Both structures are rich in ER proteins such as PDI (see Introduction), and can be labeled using the marker PDI-GFP. Lam was distributed on the NE rather than on the astral membrane at prometaphase I (Fig. 2A). Its localization along the spindle envelope structure, which is derived from the NE, as well as the astral membrane became distinctive after metaphase I (Fig. 2B,C). We quantified the anti-Lam immunostaining signal of the metaphase I cell shown in Fig. 1D using line scan software (Metamorph) and showed that a lesser amount of Lam was distributed around the spindle poles (Fig. S4). Therefore, we conclude that the B-type lamina is depolymerized at the polar regions by metaphase I and it remained to be localized along the NE, its derivative known as the spindle envelope, and the astral membrane during meiosis I.

Only around both spindle poles, the NE composed of Lam lamina seemed to break down at metaphase I. And spindle microtubules elongated from centrosomes pass into the nuclear space surrounded by the spindle envelope through the resultant holes (arrows in Fig. 1D and Fig. S4). By contrast, the anti-LamC immunostaining signal became undetectable at metaphase I (Fig. 1N). Lam was distributed along the spindle envelope structure, which displayed an oval shape, at anaphase I (Fig. 1E). As anaphase cells elongated, Lam localization was maintained on the spindle envelope surrounding the central spindle microtubules. The Lam returned to sister nuclei at telophase I and was also localized along string-like remnants of the spindle envelope linking the two sister nuclei (arrows in Fig. 1F,G). By contrast, nuclear lamina consisting of LamC, which had disappeared at metaphase I, returned to daughter nuclei at mid anaphase I, slightly earlier than Lam reassembled (Fig. 1O,P). The LamC relocalization at telophase I is also dependent on Lam as the LamC signal was found to be reduced in the cell depleted of Lam (Fig. S3D,E).

On the contrary, the Lam immunostaining signals indicated that the protein exhibited a different intracellular localization in meiosis II (Fig. 1G-J). Its immunostaining signal remained on the remnants of the spindle envelope until metaphase II (arrows in Fig. 1F,G). Except for this leftover signal, we could hardly find any anti-Lam staining around chromosomes at metaphase II (Fig. 1H), although it came back to the sister nuclei by mid anaphase II (Fig. 1I). The signal was largely detected only around sister nuclei at telophase I, except for minor signals on the remnants (Fig. 1I,J). The pattern of LamC localization at meiosis II was almost the same as it was in meiosis I (Fig. 1K-P versus Q-T). In conclusion, the breakdown of the nuclear lamina consisting of the B-type Lam differs between the first division and the second division in male meiosis.

### Lam depletion results in a failure of cytokinesis in male meiosis

This intriguing Lam localization encouraged us to examine whether the B-type Lam plays some role in the later stages of meiotic divisions. First, we looked for a spermatid phenotype that reflects some abnormality in chromosome segregation and cytokinesis during meiotic divisions. We induced expression of specific dsRNA targeted against *Lam* or *LamC* in premeiotic spermatocytes using the Gal4/UAS system and confirmed a significant depletion of each protein (Fig. S2). Surprisingly, multinuclear cells were observed in 25.2% of spermatids at the onion stage (*n*>500 spermatids from 20 males) when Lam was depleted in spermatocytes (Table S1, Fig. S6B). Abnormal spermatids with more than four nuclei were also found at a lower frequency (4.4%) in testes. These phenotypes appearing in spermatids suggest that cytokinesis was inhibited in each or both of the meiotic divisions. In contrast, such multinucleate spermatids were found in LamC-depleted testes at a much lower frequency (3.6%, *n*>500 spermatids from 20 males; Fig. S6C). Another type of abnormal spermatid in which nuclei were variable in size, indicating a defect in chromosome segregation in meiosis, was scored at a lower frequency (3.8% for Lam depletion and 1.4% for LamC depletion, *n*>500 cells from 20 males for each; Table S1). Therefore, we conclude that a reduction of Lam in spermatocytes results in a failure of cytokinesis and it also results in a defect in chromosome segregation during male meiosis less frequently.

### Lam depletion interferes maintenance of the spindle envelope structure

**Fig. 3.**
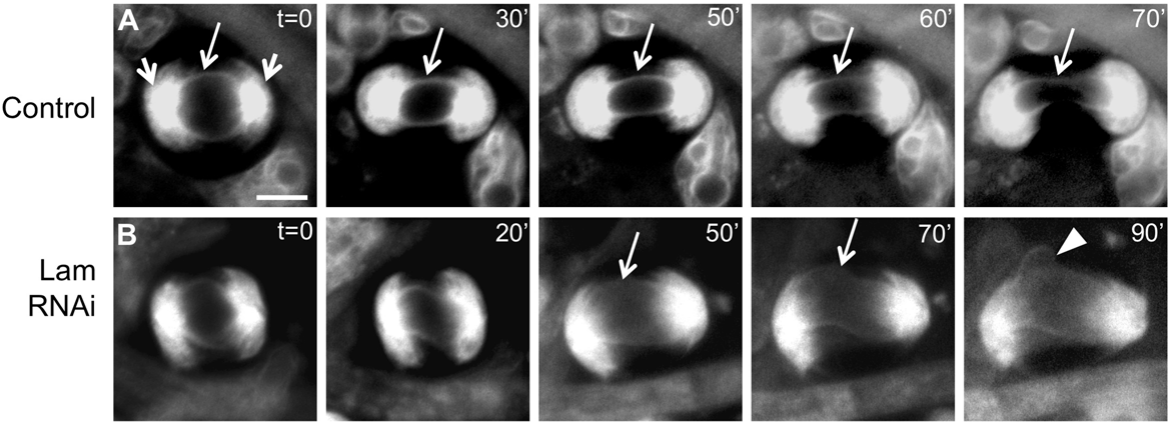
**Time-lapse observation of ER-based organelles including the NE, astral membrane and spindle envelope in a Lam-depleted spermatocyte undergoing meiosis I.** (A) A control cell. Astral membrane structures formed around both spindle poles (short arrows) at the time when chromosome congression initiated (t=0 min). The circular NE became transformed into the oval-shaped spindle envelope structure (long arrows, t=0 to t=60 min). Plasma membrane ingression then initiated (t=70 min). (B) A Lam-depleted spermatocyte undergoing meiosis I. The spindle envelope became less distinct (long arrows, t=50, 70 min), although the NE was initially indistinguishable from that of control cells (t=0 min). The spindle envelope became partially disintegrated (arrowhead) (t=90 min) and plasma membrane ingression failed to occur within the time period examined. Scale bar: 10 μm.

The distribution of Lam on the spindle envelope, which is essential for cytokinesis, prompted us to examine whether Lam contributes to the formation or maintenance of intracellular structure. We carried out time-lapse experiments to observe the dynamics of spindle envelopes labeled with PDI-GFP. In control cells, PDI fluorescence was distributed along the circular NE and on the astral membrane extending from the two polar regions at metaphase I. The NE transformed into the oval-shaped spindle envelope as the meiotic cycle progressed through anaphase I (t=30-60 min; Fig. 3A). As plasma membrane ingression proceeded at the cleavage furrow region, the spindle envelope also became constricted at the mid region (t=70 min; Fig. 3A). In contrast, we observed a thinner NE at metaphase I in Lam-depleted spermatocytes (t=0-20 min; Fig. 3B). As the cell elongated during anaphase I, the spindle envelope became unstable (t=20-50 min; Fig. 3B). The spindle envelope became hard to be recognized and the structure disintegrated at the end (t=90 min; Fig. 3B). On the other hand, the astral membrane structures at both poles were not substantially perturbed. We have reproducibly observed eight abnormal meiosis I cells displaying a similar disintegration of the spindle envelope among 17 meiotic cells examined, although we cannot exclude a possibility that it would be due to a rocking of the focal plane. We therefore conclude that Lam is required for the maintenance of the spindle envelope in male meiosis.

### Lam depletion abolishes formation of central spindle microtubules

**Fig. 4.**
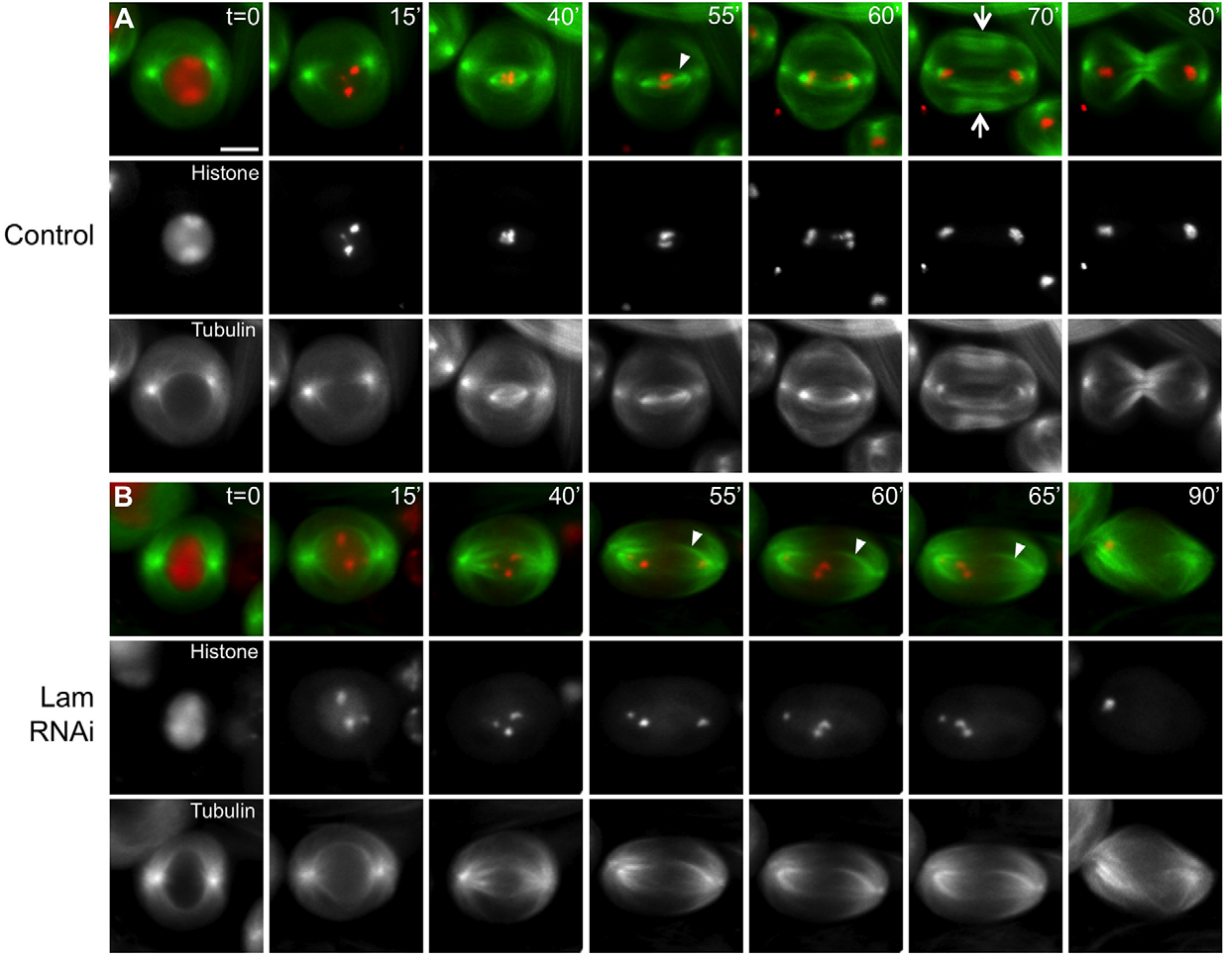
**Time-lapse observation of microtubule and chromosome dynamics in Lam-depleted spermatocytes undergoing meiosis I.** The time-lapse observation began when GFP-tubulin fluorescence became distinct (t=0 min, prophase I). Green: GFP-β-tubulin; red: histone2Av-mRFP. (A) A control cell. Astral microtubules and kinetochore microtubules formed at prometaphase I (t=40 min) and metaphase I (arrowhead, t=55 min), respectively. Robust bundles of central spindle microtubules characteristic of meiosis I were constructed at late anaphase I (arrows, t=70 min). Cytokinesis initiated and plasma membrane ingression progressed (t=70-80 min). (B) A Lam-depleted spermatocyte undergoing meiosis I. No distinct kinetochore microtubules were observed (arrowheads in B, t=55-65 min). Chromosomes failed to congress at the equator plane (t=40-65 min). Most of the chromosomes were eventually transported toward a single spindle pole (t=90 min). Cytokinesis had not initiated by the last time point (t=90 min). No bundles of central spindle microtubules were observed by the last time point (t=55-90 min). Scale bar: 10 μm.

To understand why the depletion of Lam eventually resulted in a failure of cytokinesis in male meiosis, we next examined whether the microtubule structure constructed at later stages of meiotic divisions was affected in Lam-depleted cells. We have observed nine cells containing abnormal spindle microtubules among 109 fixed samples of prometaphase I to metaphase I cells (8.3%). It was consistent with the frequency of abnormal spermatids carrying variously sized nuclei (Table S1). On the other hand, we have scored 23 cells with abnormal central spindle microtubules among 134 late anaphase I to telophase I cells (17.1%). We particularly put a focus on observation of central spindle microtubules. Next, we performed time-lapse observations of meiosis I cells (Fig. 4). In control cells, after elongation of astral microtubules from the two spindle poles (t=15 min; Fig. 4A), distinctive kinetochore microtubule bundles that brought chromosomes together to the spindle equator were constructed (t=55 min; arrowhead in Fig. 4A). The remarkable microtubule bundles, termed the central spindle microtubules, appeared at late anaphase (t=70 min; arrows in Fig. 4A). On the contrary, stable kinetochore microtubules failed to form or no central spindle microtubule formation occurred before the end of meiotic division I of the Lam-depleted cell (t=55 min and thereafter; Fig. 4B), although the elongation of astral microtubules was not affected in the meiotic cell. Interestingly, a small bundle of microtubules began to elongate but collapsed in a shorter period (t=55-90 min; arrowheads in Fig. 4B). The central spindle microtubules visible in control cells never formed in the depleted cells. We have performed time-lapse observation experiments of 15 meiosis I cells depleted of Lam and observed seven cells in which central spindle microtubules failed to be formed or maintained like the cell in Fig. 4B. Thus, we conclude that the Lam depletion interfered with central spindle formation and maintenance, which is essential for cytokinesis in male meiosis I.

### Depletion of Lam interferes with the recruitment of myosin and its assembly into a contractile ring at the cleavage furrow site

**Fig. 5.**
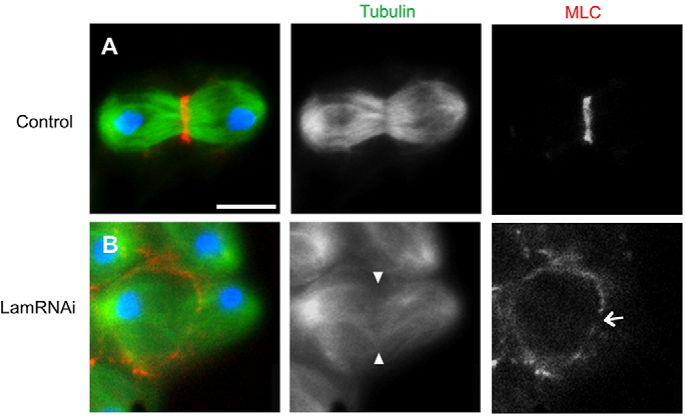
**The contractile ring component MLC fails to accumulate in Lam-depleted cells in meiosis I.** (A,B) Observation of primary spermatocytes from telophase I cysts expressing RFP-Tubulin (green) and GFP-MLC (red). Blue: DNA staining. (A) A normal telophase I cell undergoing the cleavage furrow ingression. MLC formed the contractile ring at the middle of the central spindle microtubules. (B) A primary spermatocyte from a telophase I cyst depleted of Lam. Note that the cell failed to show the furrowing. The cell possessed fewer overlapped central spindle microtubules (arrowhead) and contained the disconnected MLC ring (arrow). Lam depletion resulted in a perturbation of contractile ring formation at cleavage furrow sites. Scale bar: 10 μm.

The central spindle microtubules play an essential role in contractile ring formation at the cleavage furrow sites (see Introduction). Thus, we next examined whether depletion of Lam influences accumulation of one of the ring components, myosin light chain (MLC), at the furrow sites. In a control cell at telophase I, Lam had already localized in sister nuclei and MLC accumulated in the middle of the cell to construct the contractile ring (red for MLC at the furrow, green for microtubules in Fig. 5A). We compared this with the MLC distribution in abnormal cells contained in a telophase I cyst depleted of Lam. The MLC ring failed to be constructed at a proper position in one such telophase I-like cell possessing fewer amounts of central spindle microtubules, although the chromosomes had fully segregated (Fig. 5B). Several disconnected myosin foci were distributed along lower cell edge of the cell, suggesting that the depletion of Lam interfered with myosin ring formation. We observed 27 cells displaying abnormal MLC formation among 42 telophase I cells (from 100 males). In summary, we obtained evidences that Lam is localized on the spindle envelope in meiosis I and that its depletion affects construction of this envelope structure, the formation of central spindle microtubules and the contractile ring formation.

### Nup107 depletion affects nuclear localization of nuclear laminas consisting of Lam

As it is known that nuclear lamina interacts with several proteins of the nuclear envelope, we next examined whether NPC components influence the intracellular localization of the *Drosophila* B-type Lamin. The NPC is composed of a central core subunit containing the Nup107-160 complex, a central channel subunit, and a luminal ring and two other appendages. As a first trial, we investigated the nuclear phenotypes of premeiotic spermatocytes expressing dsRNA for each of 21 NPC components in testis cells (Fig. S5). We performed anti-Lam immunostaining to see whether spermatocytes expressing each dsRNA displayed any abnormality in the nuclear lamina (*N*>900 premeiotic spermatocytes at S5 stage from 40 males for each). Two types of nuclear lamina phenotype were scored in the premeiotic cells (Fig. S5A-C). The leakage phenotype is a less severe phenotype in which the anti-Lam immunostaining signal was also observed as small foci in the cytoplasm. In a more severe phenotype, referred to as the rupture phenotype, many pieces of nuclear lamina became detached from the nucleus and were present in the cytoplasm. In spermatocytes expressing Nup107 dsRNA, we confirmed by qRT-PCR that expression of dsRNA for Nup107 caused a 75% reduction of the mRNA in testis cells. We found that 49.3% of the cells in mature spermatocyte cysts displayed an abnormality in the nuclear lamina (the leakage phenotype appeared in 15.5% of the cells depleted of Nup107, and the rupture phenotype in 33.8% of the cells), compared to normal cells (2.6%; the leakage phenotype in 1.9% of the cells, and the rupture phenotype in 0.7% of the cells). dsRNA expression of another seven components of the Nup107-160 complex also resulted in the production of spermatocytes displaying those nuclear phenotypes (asterisks in Fig. S5D). There was a tendency that these nuclear phenotypes were observed more frequently in Nup107-160 complex protein-depleted spermatocytes than in spermatocytes expressing dsRNA for other individual NPC components (Fig. S5D).

### Nup107 co-localizes with Lam through meiosis I

**Fig. 6.**
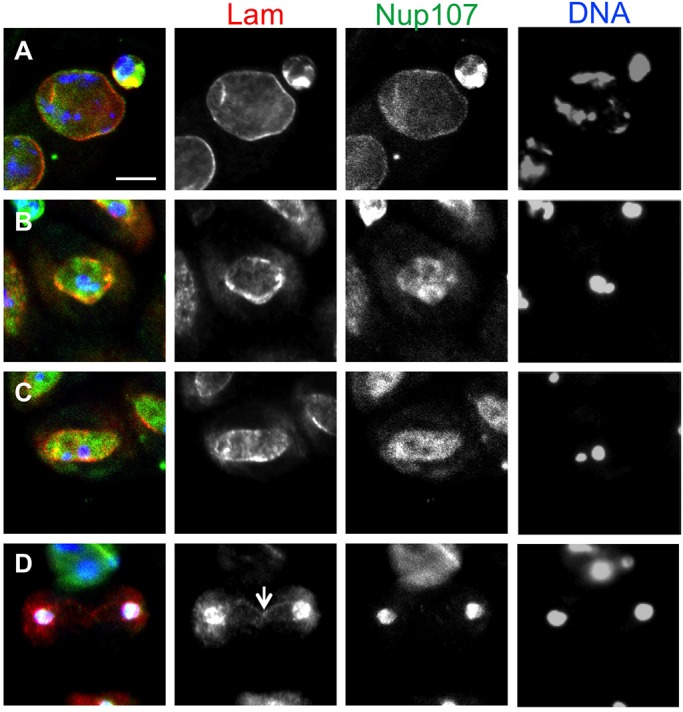
**Simultaneous observation of Lam and Nup107 in normal primary spermatocytes undergoing meiosis I in *Drosophila* males.** (A-D) Anti-Lam immunostaining (red) of primary spermatocytes expressing GFP-Nup107 (green). Their meiotic stages were speculated on the basis of a cellular localization of chromosomes (blue). (A) Two nuclei of premeiotic spermatocytes and a smaller cyst cell nucleus within a primary spermatocyte cyst. (B) Primary spermatocytes from a metaphase I to early anaphase I cyst. A round circular NE is transforming into an oval-shaped spindle envelope structure at the center of the field. (C) Spermatocytes from a late anaphase I cyst. (D) A telophase I spermatocyte. Note that Nup107 co-localizes with Lam, which is predominantly localized along the NE and the spindle envelope structure, except for minor foci on remnants of the envelope (arrow). Scale bar: 10 μm.

Having shown that the Nup107-160 complex is required for the nuclear localization of Lam, we next examined whether the Nup107-160 complex structure also influences the intracellular localization of this lamin in male meiosis. To compare the intracellular localization of Lam with that of Nup107 in meiosis, we immunostained primary spermatocytes expressing Nup107-GFP using anti-Lam antibody. Nup107 is co-localized with Lam on the spindle envelope in premeiotic nuclei (Fig. 6A). It was also found in the nuclear matrix surrounded by the NE at metaphase I (Fig. 6B) and in the space surrounded by the oval-shaped spindle envelope after anaphase I (Fig. 6C). Nup107 distribution largely overlapped with that of Lam until anaphase I. It was uniformly distributed in the space surrounded by the spindle envelope, while Lam was more concentrated on the spindle envelope. However, Nup107 was exclusively localized on sister nuclei at telophase I, while a fraction of the Lam remained localized on remnants of the spindle envelope (arrow in Fig. 6D). These localization data suggest that Lam and Nup107 can interact with each other in male meiosis. Unlike in vertebrate cells, *Drosophila* Nup107 was undetectable on the kinetochore during meiotic divisions.

### Nup107 depletion also results in a failure of cytokinesis in male meiosis

The overlapping localization of Nup107 and Lam caused us to examine whether Nup107 depletion could affect cytokinesis in meiotic divisions. Next, we observed the meiotic phenotypes that appeared in postmeiotic spermatids and spermatids with abnormally sized nuclei derived from spermatocytes expressing dsRNA for each of the NPC components. We selected at least one representative component for each NPC subunit (Nup62, Nup93, Nup107, Nup153 and Nup358) and confirmed by qRT-PCR that the dsRNA expression reduced the amount of their mRNAs to 2.6%, 13.1%, 25.0%, 35.7% and 33.6%, respectively (Fig. S7). Multinucleate spermatids were visible in the Lam-depleted testes. We calculated the average frequency of multinucleate spermatids derived from spermatocytes expressing dsRNA for each subunit of the NPC (Table S1, Fig. S6D-F). We noticed that expression of dsRNA for the Nup107-160 complex components tended to result in similar meiotic phenotypes including a production of multinucleate cells to those of Lam-depleted cells, at a modest frequency (10.4% for Nup107-depleted cells; Table S1). Our phenotypic analyses of spermatocytes expressing dsRNA for each of the 21 NPC components allow us to speculate that the Nup107-160 complex also plays a role not only in the nucleus of premeiotic spermatocytes but also in meiotic division.

### Nup107 depletion disrupts Lam localization on the spindle envelope

**Fig. 7.**
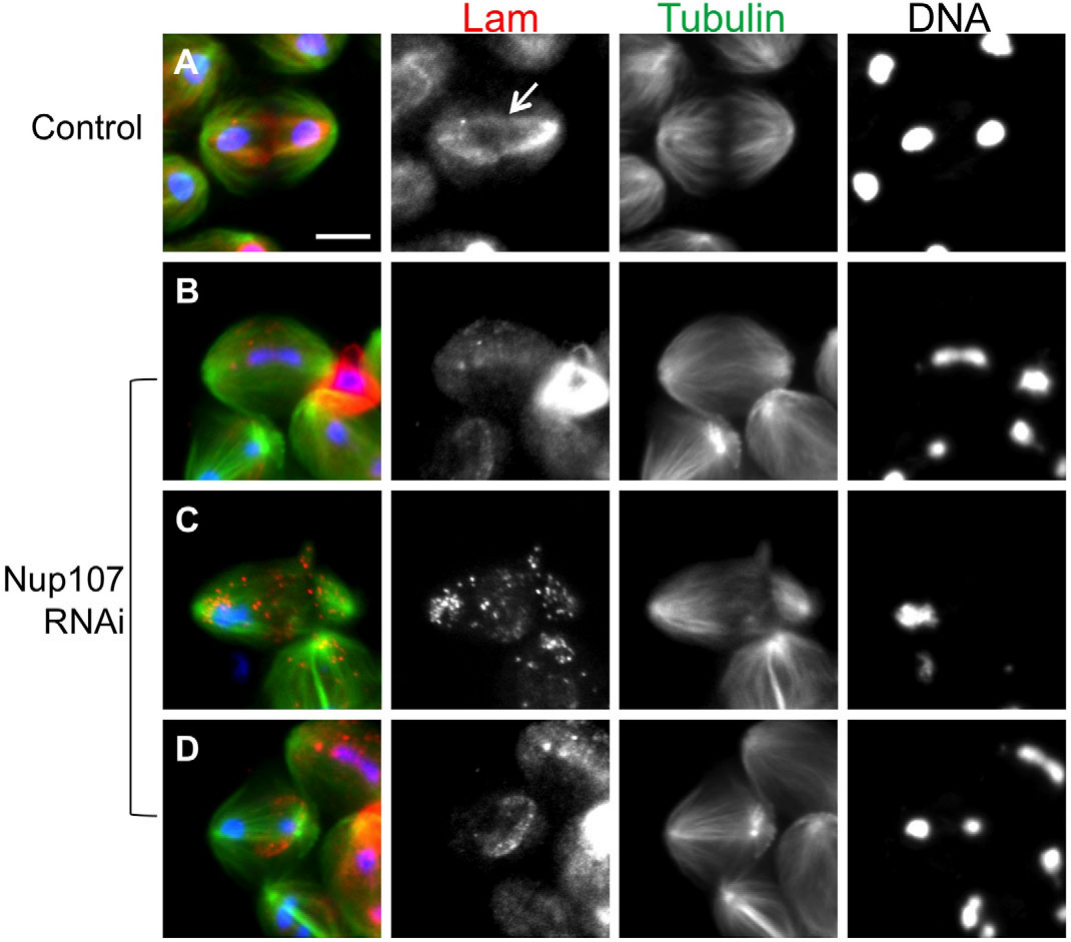
**Meiosis I cells with abnormal spindle envelopes in which Lam incorporation into the spindle envelope failed in Nup107-depleted cells.** (A-D) Anti-Lam immunostaining (red) of meiosis I cells expressing GFP-β-tubulin (green). DNA staining (blue). (A) Control anaphase I cells. Lam was localized along the spindle envelope (arrow) during meiosis I. (B-D) We have carried out multiple immunostaining experiments and observed 387 meiosis I cells from 64 males with testis specific depletion. Three types of abnormal cells were observed in anaphase I to telophase I cysts depleted of Nup107. (B) A weaker anti-Lam immunostaining signal appears around segregating chromosomes at early anaphase I in the depleted cells, although a considerable amount of Lam can be detected in cyst cells within the same cyst. (C) A meiotic cell (center) from a late anaphase I cyst showing the Lam signal dispersed through the cytoplasm as many small foci. (D) The Lam is distributed on an abnormally short spindle envelope, partially formed associated with segregating chromosomes. Chromosomes segregating toward the left spindle pole protruded out of the nuclear space surrounded by the Lam lamina (red). Scale bar: 10 μm.

As Nup107 co-localized with the B-type lamin on the NE at premeiotic stage as well as on the spindle envelope derived from the NE during meiosis I, we finally examined whether Lam distribution was affected in Nup107-depleted cells. In control cells, the lamin was distributed along the spindle envelope structure in anaphase I cells (Fig. 7A). In contrast, a weaker anti-Lam immunostaining signal was observed around segregating chromosomes at earlier anaphase I in the Nup107-depleted cells (Fig. 7B). Ten abnormal cells displaying such a phenotype have been scored among 42 anaphase I cells examined. Some meiotic cells displayed a Lam signal dispersed as many small foci through the cytoplasm (Fig. 7C; 14/42 cells). In another anaphase cell, the signal was distributed on an abnormally shorter spindle envelope that was partially formed in the middle of the cells between segregating chromosomes (Fig. 7D; 5/42 cells). Therefore, we conclude that the depletion of Nup107 also disrupts the integrity of the spindle envelope on which Lam is localized in meiosis I.

## DISCUSSION

We investigated whether a B-type lamin (Lam), an A-type lamin (LamC) and nuclear pore complex components play a certain role in *Drosophila* male meiotic divisions. Our immunostaining experiments and PLA *in situ* analyses revealed that these two lamins are closely associated with each other in the nuclear lamina of premeiotic spermatocytes and in the regenerated sister nuclei at telophase. LamC nuclear localization was dependent on Lam localization, but not vice versa. Interestingly, Lam displayed different cellular localization during meiotic divisions I and II. Lam along the NE-derived spindle envelope continued to be localized on this structure at metaphase I and thereafter. The lamin returned to sister nuclei at late anaphase. On the contrary, nuclear lamina consisting of LamC disassembled completely at metaphase I and LamC returned to daughter nuclei at mid anaphase I. Additionally, neither of the A-type and B-type nuclear lamins was detected in the cells at metaphase II. That is, the breakdown of nuclear lamina consisting of the B-type lamin differs between first and second division in male meiosis. We also examined meiotic phenotypes that appeared in postmeiotic spermatids derived from cells depleted of either Lam or LamC. Surprisingly, Lam depletion resulted in a failure of cytokinesis in one or both meiotic divisions and in a defect in meiotic chromosome segregation less frequently. In contrast, abnormal meiotic phenotypes were found in LamC-depleted testes at a much lower frequency. Our time-lapse observation of the primary spermatocytes showed that depletion of the B-type lamin perturbed the formation and maintenance of spindle envelope structure. We further showed that Lam depletion affected the formation of central spindle microtubules, which are known to be essential intracellular structures for the initiation of cytokinesis in male meiosis. The depletion affected recruitment of a contractile ring component to cleavage sites. Furthermore, we examined whether NPC components also have similar meiotic functions. Nup107, which is a core scaffold protein of the NPC, is co-localized with Lam through meiosis I. Nup107 depletion affected nuclear localization of the Lam-containing lamina, and resulted in similar meiotic phenotypes such as a failure of cytokinesis in meiotic divisions. Finally, the depletion disrupted Lam localization on the spindle envelope. Nup107 depletion therefore influenced meiotic cytokinesis by perturbing Lam localization.

### A-type and B-type lamin differ in their dynamics and functions in male meiotic divisions

*Drosophila* cells have two types of nuclear lamins; Lam corresponds to B-type lamin and is expressed ubiquitously, while the A-type lamin, LamC, is expressed in a tissue-specific manner (Riemer et al., 1995; Schulze et al., 2009). Lam contains a CAAX motif that is required for binding to the inner nuclear membrane, whereas LamC does not. In this study, we showed that these two types of lamins are associated with each other at a distance sufficiently close for them to be contained in the same complex. The nuclear localization of LamC is dependent on Lam, but not vice versa. Therefore, it is possible to speculate that a nuclear lamina consisting of Lam plays a role as a major scaffold component that is directly connected with the nuclear membrane through its CAXX motif. These results are consistent with previous data from vertebrate cells indicating that heterotypic interactions between A- and B-type lamins are present and that lamin co-assembly is potentially important for the formation of whole nuclear laminas (Delbarre et al., 2006; Schirmer et al., 2001). It was reported that depletion of lamin B induced apoptosis but that of lamin A failed to affect cell growth in mammalian cultured cells (Harborth et al., 2001). The B-type lamin is considered to have a more important role related to mitosis. It is localized in the polar regions of the spindle matrix, and its depletion from *Xenopus* mature oocyte extracts resulted in abnormal formation of spindle microtubules (Ma et al., 2009). B-type lamin is also essential for mitosis in *C. elegans* (Liu et al., 2000). By contrast, a fewer reports have described the involvement of A-type lamin in cell division. Although LamC is also essential for *Drosophila* development (Furukawa et al., 2009; Schulze et al., 2009), its depletion caused no significant defects in the NE of premeiotic spermatocytes. LamC may play a supplementary role that supports the mesh structure of the Lam nuclear lamina in the primary spermatocytes.

Although Lam and LamC associate closely with each other to construct the nuclear lamina structure in premeiotic cells, these two lamins showed a different intracellular distribution during meiotic division I. The LamC lamina was completely decomposed before metaphase I and its components dispersed through the cytoplasm, while Lam was distributed on the spindle envelope structure surrounding the space in which chromosomes are segregated during meiosis I. By contrast, in meiosis II, both laminas were almost completely disassembled prior to metaphase II. Lam may play a role in strengthening the spindle envelope in meiosis I. This envelope structure is rich in ER proteins and is required for the formation of spindle microtubules that are closely associated with the envelope structure (Kitazawa et al., 2012). During *Drosophila* development, two types of NEBD are programmed; semi-open mitosis is observed at syncytial blastoderm stages and in larval neuroblasts (Katsani et al., 2008), while open mitosis is typical after cellularization, as shown in S2 cultured cells. As the nucleus in male meiosis I cell is surrounded by multiple layers of membranes including the NE and other related membranes termed parafusorial membranes (Fuller, 1993, see Introduction), it is also classified into semi-open type cell division. In open mitosis, most of the NE components move toward the ER or are dispersed throughout the cytoplasm after NEBD (Puhka et al., 2007). Therefore, the nuclear proteins involved in chromosome condensation or kinetochore function are likely to be dispersed and diluted within a large proliferative cell such as the primary spermatocyte. It would be advantageous for the execution of efficient cell divisions to maintain nuclear proteins at a higher concentration within the limited cell space enclosed by the spindle envelope. Therefore, we can also consider the same explanation for semi-open mitosis in larval neuroblasts of a larger size (Katsani et al., 2008). Another previous study argued that the spindle envelope structure is useful to prepare the nuclear membrane components necessary for the rapid reassembly of NEs at telophase I (Groigno and Whitaker, 1998). However, LamC returned to the sister nuclei earlier than did Lam, albeit it was disassembled completely at metaphase I. Few reports have so far described NEBD in *Drosophila* male meiosis II. We have not found the spindle envelope structure in secondary spermatocytes; they display complete NEBD in meiosis II, as observed in mitotic culture cells. As the secondary spermatocytes are much smaller than the primary cells, they may not need to retain division factors in the isolated intracellular space.

### Lam facilitates formation and maintenance of the spindle envelope structure required for cytokinesis in *Drosophila* male meiosis

Lam is localized on the nuclear envelope from premeiotic stages to metaphase I. As the primary spermatocyte elongated thereafter, multilayer membranes surrounding the nucleus transformed from a round to an oval shape, termed the spindle envelope (Debec and Marcaillou, 1997; Kitazawa et al., 2012; Stafstrom and Staehelin, 1984). Lam continued to be localized on the spindle envelope at metaphase I and thereafter. It became associated with chromatin in sister nuclei again after late anaphase I. Lam seemed to be tightly associated with the spindle envelope, as a fraction of the protein still remained localized on remnants of the spindle envelope in meiosis II. In addition to its localization on the spindle envelope until the later stage of meiosis I, the meiotic phenotypes of cells depleted of Lam suggest that nuclear lamin could influence cytokinesis in male meiotic divisions (Table S1). Although there is little evidence that nuclear lamins are related to cytokinesis in cell division, it has been shown that B-type lamins are localized in the spindle matrix, which is closely associated with spindle microtubules, in *Xenopus* oocyte extracts and in Hela cells (Smythe et al., 2000; Tsai et al., 2006). These authors also showed that inhibition of B-type lamins by expression of its dominant negative form resulted in the formation of abnormal spindle microtubules. These data indicate that lamin B can influence construction of the spindle matrix and spindle microtubules. However, it has not been reported whether the observed abnormal spindle microtubules influenced later stages of cell division such as cytokinesis. By contrast, our time-lapse observation of microtubules in primary spermatocytes showed that the lamin depletion disrupted the formation and maintenance of the characteristic microtubule bundles that appear at late anaphase I. These microtubules, which overlap at their plus ends, are termed the central spindle microtubules (Fuller, 1993; Bucciarelli et al., 2003; Giansanti et al., 1998; Inoue et al., 2004). Previous studies reported that these characteristic microtubules are required for the initiation of cytokinesis by mediating contractile ring formation (Giansanti et al., 1998; Jang et al., 2005; Verni et al., 2004). Another study reported that the spindle envelope plays an essential role in facilitating the formation and maintenance of central spindle microtubules at late anaphase I (Kitazawa et al., 2012). Therefore, it is possible that Lam facilitates the formation and reinforcement of the spindle envelope that supports formation of the central spindle microtubules. It is also possible to speculate that the disruption of the intracellular structure by Lam depletion may result in a failure of cytokinesis.

### Nup107-160 complex influences cytokinesis by ensuring that Lam is localized on the spindle envelope in *Drosophila* male meiosis

Nuclear proteins such as lamins are transported into the nucleus through NPCs. Nup107 plays an important role as a core scaffold protein of the NPC (Antonin et al., 2008; Walther et al., 2003). We showed that depletion of Nup107 and dsRNA expression of other components of the Nup170-160 complex resulted in the precocious release of nuclear lamina consisting of Lam into the cytoplasm. It is likely that Nup107 depletion has a substantial effect on the entire NPC structure such as leaving holes in the nuclear membrane. A close interaction between nuclear lamina and NPCs was elucidated from studies of *Drosophila*, *C. elegans* and mammalian cultured cells (Lenz-Bohme et al., 1997; Liu et al., 2000; Maeshima et al., 2006). Thus, the disintegrated lamina phenotype of premeiotic spermatocytes depleted of the Nup107-160 complex may reflect the interaction between lamins and NPCs in the NE.

Nup107 is also required for the proper formation of spindle microtubules (Loiodice et al., 2004), and is thought to be a component of the spindle matrix (Katsani et al., 2008). In this study, we showed that Nup107 is co-localized with Lam along the NE as well as its derivative, spindle envelope. And we demonstrated that its depletion resulted in a disintegration of nuclear lamina as well as in a similar cytokinesis phenotype in male meiosis. We can speculate that the Nup107 plays an important role in the localization of Lam on the spindle envelope. The depletion or dsRNA expression of each component of Nup93 complex also resulted in a production of multinucleate cells at similar frequencies as that in Nup170-depleted cells (22% of the spermatids scored). In the NPC, the spoke complex subdomain comprising the Nup93 complex components is adjacent to the cytoplasmic ring containing Nup107 in the central core of the NPC (Powers and Dasso, 2004). Therefore, it is possible to speculate that the Nup93 depletion interfered with integrity of the B-type lamina possibly through perturbation of the NPC scaffold, and that it can eventually lead to the cytokinesis phenotype. On the other hand, other review argued the interaction between lamins and NPCs may play a less direct role in NE construction or nuclear-cytoplasmic transport in animal and plant cells (Fiserova and Goldberg, 2010). However, some evidence implying a relationship between B-type lamin and the NPC has been reported from studies of mammalian cultured cells and *Drosophila* eye development (Lenz-Bohme et al., 1997; Smythe et al., 2000). Thus, depletion or dsRNA expression of the NPC components in this study may have also eventually led to a failure of cytokinesis through inhibition of lamin function on the spindle envelope.

Furthermore, recent studies have demonstrated that some Nups themselves play critical mitotic roles related to spindle formation (Gonzalez-Aguilera and Askjaer, 2012; Nakano et al., 2010; Wong et al., 2006). The Nup107-160 complex is required for correct bipolar spindle assembly through interaction with the spindle checkpoint protein, Mad1 (Gonzalez-Aguilera and Askjaer, 2012; Orjalo et al., 2006). It is also possible to interpret the mitotic phenotype of the Nup-depleted cells as some Nups themselves have mitotic roles related to spindle envelope formation.

Moreover, one could consider another possibility to explain the cytokinesis phenotype of cells depleted of the NPC components. The depletion or dsRNA expression of the cytoplasmic filament components (Nup214 and Nup358) also resulted in a failure of cytokinesis (Table S1). In these cases, a serious leakage of nuclear lamins had not been observed (Fig. S5). It was reported that the cytoplasmic filaments play a role as footholds for centrosome migration along the NE (Splinter et al., 2010). If centrosome localization would be perturbed in cells depleted Nup358, it could abolish the spindle formation (Riparbelli et al., 2002; Sitaram et al., 2012; Wakefield et al., 2001). Therefore, it is conceivable that mis-localization of the spindle poles occurred by depletion of the cytoplasmic filament components could also eventually lead to a failure of cytokinesis in *Drosophila* male meiosis.

## MATERIALS AND METHODS

### Drosophila stocks

*y w*, or *βtubulin-GFP* stocks that have been previously described (Inoue et al., 2004) were used as a normal control for cytological studies and time-lapse observations. The following stocks containing UAS-RNAi constructs for two lamin genes and 21 NPC genes were obtained from Vienna Drosophila RNAi Center (Czech et al., 2013; Muerdter, et al., 2013; Mummery-Widmer et al., 2009; Neumüller et al., 2011), Bloomington Stock Center and National Institute of Genetics, Mishima (Table S2). We used *UAS-dir2*; *bam-Gal4::VP16* for spermatocyte-specific dsRNA expression and *UAS-GFP* RNAi as a negative control. A protein trap stock expressing GFP-PDI was obtained from L. Cooley (Yale University, New Heaven, USA). *LamC^G00158^* expressing GFP-LamC was provided from L. Wallrath (University of Iowa, Iowa City, USA). *mRFP-Nup107^7.1^* (Katsani et al., 2008) was a gift from V. Doyle (Institut Jacques Monod, Paris, France).

### Western blot analysis

Western blots to detect Lam and LamC were incubated with antibodies against ADL84.12 and LC28.26 [Developmental Studies Hybridoma Bank (DSHB), Iowa, USA], respectively, and followed by incubation with HRP-conjugated anti-mouse IgG.

### Quantitative real-time PCR analysis

qRT-PCR analysis was performed to determine the expression level of lamin genes and Nups genes in adult flies with induced expression of each dsRNA with a *bam-Gal4* driver as described (Yasukawa et al., 2015). Total RNA was extracted from adult flies using the TRIzol reagent (Invitrogen, Carlsbad, CA, USA). cDNA synthesis from the total RNA was carried out using a PrimeScript II High Fidelity RT-PCR kit (Takara, Shiga, Japan) with random primers. qRT-PCR was performed using FastStart Essential DNA Green Master (Roche, Mannheim, Germany) and a LightCycler Nano (Roche, Basel, Switzerland). *RP49* was used as a normalization reference (Oka et al., 2015). Relative mRNA levels were quantified using LightCycler Nano software version 1.0 (Roche, Basel, Switzerland).

### Live cell imaging of primary spermatocytes

To observe cytokinesis in living primary spermatocytes more easily, we added some modifications to the protocol described previously (Inoue et al., 2004; Kitazawa et al., 2012, 2014). To examine dynamics of the NE and its derivatives in male meiosis, flies expressing GFP-PDI were used. Testes from the adult flies were dissected and cells were laid out under mineral oil (Trinity Biotech, Bray, Ireland) in open chambers surrounded by double-faced tape on clean glass coverslips without any pressure. Time-lapse imaging was performed on an Olympus IX81 fluorescence microscope (Olympus, Tokyo, Japan) outfitted with excitation, emission filter wheels (Olympus, Tokyo, Japan). Cells were imaged with a 40× lens. At each 30-s time interval, near-simultaneous GFP and/or RFP fluorescence images were captured with a CCD camera (Hamamatsu Photonics, Shizuoka, Japan). Image acquisition was controlled through the Metamorph software package running on a PC. Testis squashes to evaluate onion-stage spermatids were made using previous protocols (Inoue et al., 2004) and viewed by phase contrast microscopy.

### Immunofluorescence

Testis cells were fixed according to the method of Inoue et al. (2004). For immunostaining, anti-Lam antibody (ADL84.12, DSHB) was used at a 1:200 dilution. Microtubules were visualized by immunostaining with anti-αTubulin (DM1A, Sigma-Aldrich, St Louis, MO, USA) or expression of GFP-βTubulin (Inoue et al., 2004). All secondary antibodies and DNA stains were commercially obtained. LamC was visualized by expression of GFP-LamC. ER-based structures such as the NE and its derivatives were visualized by expression of GFP-PDI residing predominantly in the intracellular structure. Images were processed and merged in pseudocolor using Metamorph version 7.6 (Molecular Devices, Sunnyvale, CA, USA).

### PLA *in situ* method

*In situ* PLA that enables detection of protein interaction within cells were performed according to the Duolink kit method (Nacalai Inc., Kyoto, Japan). We applied the *in situ* PLA with a combination of antibodies that allowed us to detect close association between Lam and LamC. We used ADL84.12 antibody (DSHB, Iowa, USA) to recognize Lam, and anti-GFP (ab290, Abcam, Tokyo, Japan) for GFP-LamC detection.

### Live cell imaging of primary spermatocytes

We followed a protocol as described previously (Inoue et al., 2004; Kitazawa et al., 2014). Testes from adult flies were dissected and spread under mineral oil (Trinity Biotech plc.) on clean glass cover slips in open chambers surrounded by double-faced tape. Time-lapse imaging was performed using an Olympus IX81 fluorescence microscope equipped with excitation and emission filter wheels (Olympus, Inc., Tokyo, Japan). For each 30-s interval, GFP fluorescence images or GFP and RFP fluorescence images were sequentially captured with a CCD camera (Hamamatsu Photonics, Shizuoka, Japan). Image acquisition was controlled by Metamorph software (Molecular Devices, CA, USA). Testes squashes to evaluate onion-stage spermatids were performed according to a protocol as described previously (Ichihara et al., 2007; Inoue et al., 2004; Kitazawa et al., 2014) and viewed under phase-contrast microscopy.
